# Variation in *Trans*-3′-Hydroxycotinine Glucuronidation Does Not Alter the Nicotine Metabolite Ratio or Nicotine Intake

**DOI:** 10.1371/journal.pone.0070938

**Published:** 2013-08-02

**Authors:** Andy Z. X. Zhu, Qian Zhou, Lisa Sanderson Cox, Jasjit S. Ahluwalia, Neal L. Benowitz, Rachel F. Tyndale

**Affiliations:** 1 Department Pharmacology and Toxicology, University of Toronto, Ontario, Canada; 2 Department of Preventive Medicine and Public Health, University of Kansas School of Medicine, Kansas City, Kansas, United States of America; 3 Department of Medicine and Center for Health Equity, University of Minnesota Medical School, Minneapolis, Minnesota, United States of America; 4 Division of Clinical Pharmacology and Experimental Therapeutics, Departments of Medicine and Bioengineering & Therapeutic Sciences, University of California San Francisco, San Francisco, California, United States of America; 5 Campbell Family Mental Health Research Institute, Center for Addiction and Mental Health and Department of Psychiatry, University of Toronto, Ontario, Canada; Imperial College London, United Kingdom

## Abstract

**Background:**

CYP2A6 metabolizes nicotine to its primary metabolite cotinine and also mediates the metabolism of cotinine to trans-3′-hydroxycotinine (3HC). The ratio of 3HC to cotinine (the “nicotine metabolite ratio”, NMR) is an *in vivo* marker for the rate of CYP2A6 mediated nicotine metabolism, and total nicotine clearance, and has been associated with differences in numerous smoking behaviors. The clearance of 3HC, which affects the NMR, occurs via renal excretion and metabolism by UGT2B17, and possibly UGT2B10, to 3HC-glucuronide. We investigated whether slower 3HC glucuronidation alters NMR, altering its ability to predict CYP2A6 activity and reducing its clinical utility.

**Methods:**

Plasma NMR, three urinary NMRs, three urinary 3HC glucuronidation phenotypes and total nicotine equivalents were examined in 540 African American smokers. The *UGT2B17* gene deletion and UGT2B10*2 were genotyped.

**Results:**

The *UGT2B17* gene deletion, but not UGT2B10*2 genotype, was associated with slower 3HC glucuronidation (indicated by three 3HC-glucuronidation phenotypes), indicating its role in this glucuronidation pathway. However, neither lower rates of 3HC glucuronidation, nor the presence of a *UGT2B17* and *UGT2B10* reduced function allele, altered plasma or urinary NMRs or levels of smoking.

**Conclusions:**

Variation in 3HC glucuronidation activity, including these caused by *UGT2B17* gene deletions, did not significantly alter NMR and is therefore unlikely to affect the clinical utility of NMR in smoking behavior and cessation studies. This study demonstrates that NMR is not altered by differences in the rate of 3HC glucuronidation, providing further support that NMR is a reliable indicator of CYP2A6 mediated nicotine metabolism.

## Introduction

There are currently more than 1.3 billion tobacco smokers worldwide. Nicotine is the primary psychoactive tobacco component responsible for tobacco dependence. The rate of nicotine clearance is variable, and is associated with the level of tobacco consumption [1]–[3]. In humans, the majority of nicotine is metabolized to cotinine relatively quickly (nicotine’s half-life is around 2 hours and the *in vivo* total body clearance is 1.0–1.5 L/min) by a genetically polymorphic enzyme CYP2A6 [4], [5]. Cotinine is further metabolized by CYP2A6 to trans-3′-hydroxycotinine (3HC) at a relatively slower rate (cotinine’s half-life is around 12–15 hours and the *in vivo* total body clearance is 0.05 L/min) [5]–[7]. The disposition of nicotine was previously reviewed in detail with indicative diagrams by [8], [9]. The ratio of 3HC/cotinine (also known as the nicotine metabolite ratio or NMR), is used as an *in vivo* biomarker of CYP2A6 activity, and correlates highly with nicotine clearance [10]. A number of studies have demonstrated that smokers with faster CYP2A6 activity and higher NMR have higher tobacco consumption [11], lower odds of smoking cessation without any active pharmacological intervention [12], [13], and lower odds of smoking cessation from transdermal nicotine patch therapy [14]. In humans, cotinine has a much longer half-live compared to 3HC (16 hours vs. 5 hours, respectively) [15], [16], and at steady state the elimination rate of 3HC is essentially formation-limited. Due to these pharmacokinetic properties, among regular smokers the NMR is generally stable over time and highly reproducible [17], [18]. However, the steady state level of 3HC is determined by both the rate of 3HC formation and the rate of 3HC clearance. Thus, a slow rate of 3HC clearance could increase plasma 3HC levels for a given cotinine level, resulting in a higher NMR compared to those with normal rates of 3HC clearance. This effect on NMR is not related to nicotine clearance or CYP2A6 activity and could reduce the clinical utility of NMR. The influence of variation in 3HC clearance on NMR is the subject of current investigation.

In humans, the majority of 3HC is cleared renally without further metabolism [8]. However, a fraction of 3HC can be O-glucuronidated by UDP-Glucuronosyltransferase 2B17 (UGT2B17), and other members of the UGT enzyme family, to form 3HC-*O*-Glucuronide [19]. The human *UGT2B17* gene is polymorphic; the *UGT2B17* gene deletion (sometimes known as UGT2B17*2) occurs at allele frequencies of ≈30% in Caucasians, ≈25% in African Americans, and ≈80% in Asians [20]. Hence, approximately 50%, 45% and 95% of Caucasians, African Americans and Asians have at least one copy of *UGT2B17* gene deletion respectively. The *UGT2B17* gene deletion has been associated with altered metabolism of UGT2B17 substrates including 3HC, 4-(methylnitrosamino)-1- (3-pyridyl)-1-butanol (NNAL) and testosterone [21]–[23]. Recently, it has been hypothesized that 3HC clearance might be slower in the individuals with *UGT2B17* gene deletions, which might result in higher plasma 3HC levels for a given amount of 3HC formation from cotinine by CYP2A6 [19]. Consistent with this possibility, Alaska Native people, who have a high prevalence of *UGT2B17* deletion allele like Asians (Zhu and Tyndale, unpublished observation), had a higher average NMR compared with African Americans who have a lower prevalence of *UGT2B17* deletion allele [24]. Thus, it is important to clarify this potential source of variation in glucuronidation on NMR.

In addition to the O-glucuronidation pathway, 3HC can be N-glucuronidated in human liver microsomes by UGT2B10 on the nitrogen of the pyridine ring [19]. However, 3HC-*N*-glucuronide has not been detected *in vivo* [19], [25], [26]. The human *UGT2B10* gene is polymorphic. UGT2B10*2 (rs61750900, Asp67Tyr) was previously associated with reduced UGT2B10 activity [27], [28]. Here we examine the impact of the *UGT2B17* gene deletion and UGT2B10*2 genotype on the rate of 3HC glucuronidation, NMR and tobacco consumption.

## Methods

### Study Description

The impacts of *UGT2B17* and *UGT2B10* genotype on 3HC glucuronidation and NMR were assessed in 540 treatment-seeking African American smokers as previously described [29]. Briefly, eligible participants self-identified as “African American” or “Black”, were at least 18 years old, had smoked 10 or fewer cigarettes per day for at least 6 months prior to enrollment, and smoked on at least 25 of last 30 days. Plasma samples were available for all the subjects, DNA samples were available for 531 subjects, and urine samples were available for 429 of these subjects. Nicotine dependence was measured using the Fagerstrom Test for Nicotine Dependence (FTND). All participants provided written informed consent in accordance with the principles expressed in the Declaration of Helsinki, and the study protocol was approved by the University of Kansas Human Subject Committee, the University of Toronto Ethics Review Office, and the University of California San Francisco Human Research Protection Program.

### Genotyping

*UGTB17* gene deletion and UGT2B10*2 were genotyped using ABI Viia 7 real time PCR machine (Applied Biosystems, Foster City, CA). The *UGT2B17* genotyping reaction was performed with 5 µL TaqMan GTXpress master mix and 5 µL of water containing 10 ng of DNA and 0.5 µL of 20x Taqman copy number variant genotyping assay (Hs03185327_cn, specific target *UGT2B17* exon 1 which does not amplify the pseudogene *UGT2B15*, Applied Biosystems, Foster City, CA). The UGT2B10*2 genotyping reaction was performed with 5 µL TaqMan GTXpress master mix and 5 µL of water containing 10 ng of DNA and 0.25 µL of 40x Taqman genotyping assay (rs61750900, Applied Biosystems, Foster City, CA) [22]. The reactions were performed in 96 well plates. The allele discrimination data were analyzed by Viia 7 software version 1.2.

### Analytical Chemistry

Plasma cotinine and 3HC levels were determined using liquid chromatography–tandem mass spectrometry as described previously [10]. The urinary analytes were determined using liquid chromatography–tandem mass spectrometry as described previously [30]. Urinary total nicotine equivalents (TNE) was defined as the total urinary level of nicotine and nine of its metabolites (i.e. nicotine, nicotine glucuronide, cotinine, cotinine glucuronide, 3HC, 3HC glucuronide, nicotine-*N*-oxide, cotinine-*N*-oxide, nornicotine and norcotinine). The ten analytes account for about 90% of nicotine dose [31], and creatinine adjusted spot urinary TNE correlates with daily tobacco consumption [32].

### 3HC Glucuronidation Phenotypes

Here, we used 3HC-Gluc over 3HC-free ratio (i.e. the ratio of product over substrate) as the primary 3HC glucuronidation phenotype. The percentage of TNE excreted as 3HC-Gluc and the percentage of total 3HC excreted as 3HC-Gluc were used as secondary 3HC glucuronidation phenotypes.

### Plasma and Urinary NMR Definition

Plasma NMR was defined as the ratio of 3HC over cotinine. The primary urinary NMR was defined as the ratio of total 3HC (free and glucuronide conjugated) over free cotinine since only free cotinine is available to be metabolized to 3HC. The ratio of free 3HC over free cotinine (i.e. non-glucuronidated) and the ratio of total 3HC over total cotinine (i.e. total of glucuronidated and non-glucuronidated) were used as secondary urinary NMR phenotypes for comparison to the literature [33], [34]. We obtained very similar results between all three versions of urinary NMR.

### Statistical Analyses

Comparisons of 3HC-glucurondiation phenotypes or NMRs for *UGT2B17* or *UGT2B10* genotypes were performed by Kruskal-Wallis, Mann-Whitney or Chi^2^ tests. Post-hoc adjustments were done by Dunn’s multiple comparison tests. The correlations between the primary 3HC glucuronidation phenotype with plasma NMR, urinary NMR, plasma cotinine or urinary TNE were assessed by Spearman’s correlation. Statistical analyses were performed using Stata 11 (StataCorp, College Station, TX).

## Results

### *UGT2B17* and *UGT2B10* Genotyping

The *UGT2B17* gene deletion and UGT2B10*2 were both in Hardy-Weinberg equilibrium with allele frequencies of 23.4% and 4.3% in this population of African Americans respectively (HWE P = 0.36 and 0.99 respectively) consistent with published frequencies [20], [28]. No significant difference in baseline demographics, smoking behaviors, baseline plasma cotinine levels, urinary TNE, and levels of nicotine dependence were observed between *UGT2B17* or *UGT2B10* genotype groups (Table 1), indicating that neither the *UGT2B17* gene deletion nor the UGT2B10*2 were associated with the levels of tobacco consumption or nicotine dependence.

**Table 1:** Impacts of genetic variation in *UGT2B17* and *UGT2B10*.

	*UGT2B17*				*UGT2B10* 2			
	WT/WT	WT/−	−/−	*P*-value	*1/*1	*1/*2	*2/*2	*P*-value
Number of participants	315	183	33		490	44	1	
Sex (% Female)	67.3%	63.4%	60.6%	0.56	66.3%	61.4%	0	0.31
Age	46.5	46.4	46.9	0.96	46.8	43.8	54	0.19
(Years)	(45.2–47.8)	(44.8–47.9)	(43.2–50.5)		(45.7–47.8)	(40.6–46.9)	(N/A)	
Menthol (% Smoke mentholated cigarettes)	83.8%	83.6%	81.8%	0.96	82.7%	90.9%	100%	0.35
Baseline cigarettes per day	8.1	7.7	7.7	0.27	8.0	7.6	1	0.10
	(7.8–8.4)	(7.4–8.1)	(6.6–8.7)		(7.8–8.2)	(6.8–8.4)	(N/A)	
Plasma Cotinine	278	271	273	0.89	273	281	631	0.26
(ng/mL)	(260–295)	(248–294)	(214–332)		(260–286)	(221–342)	(N/A)	
Urinary total nicotine equivalent^1^	61.9	53.2	52.1	0.30	59.1	49.0	N/A	0.54
(nmol/mg Cre)	(53.6–70.2)	(47.1–59.2)	(37.7–66.4)		(53.3–64.9)	(33.5–64.5)		
Fagerstrom Test for Nicotine Dependence	3.2	3.1	3.0	0.51	3.2	2.9	4	0.58
(FTND)	(3.0–3.4)	(2.8–3.3)	(2.4–3.7)		(3.0–3.3)	(2.4–3.4)		
**3HC-Glu/Total 3HC** 1	**19.1%**	**15.3%**	**8.0%**	**<0.0001**	17.4%	15.9%	N/A	0.53
	**(17.4%–** **20.7%)**	**(13.3%–** **17.3%)**	**(2.0%–** **14.2%)**		(16.1%–18.7%)	(11.4%–20.5%)		

Data presented as Mean (95% Confident Interval).

1 The urinary total nicotine equivalents and urinary 3HC-Glu/total 3HC ratios were only available for 426 individuals.

2 No statistically significant difference was observed in any of the demographic variables when the UGT2B10*2/*2 group was pooled with the *1/*2 group. Statistical comparison performed by Mann-Whitney or Kruskal-Wallis tests.

### *UGT2B17* Gene Deletion was Associated with Slower 3HC-glucurondation Phenotypes *in vivo*

**Figure 1:**
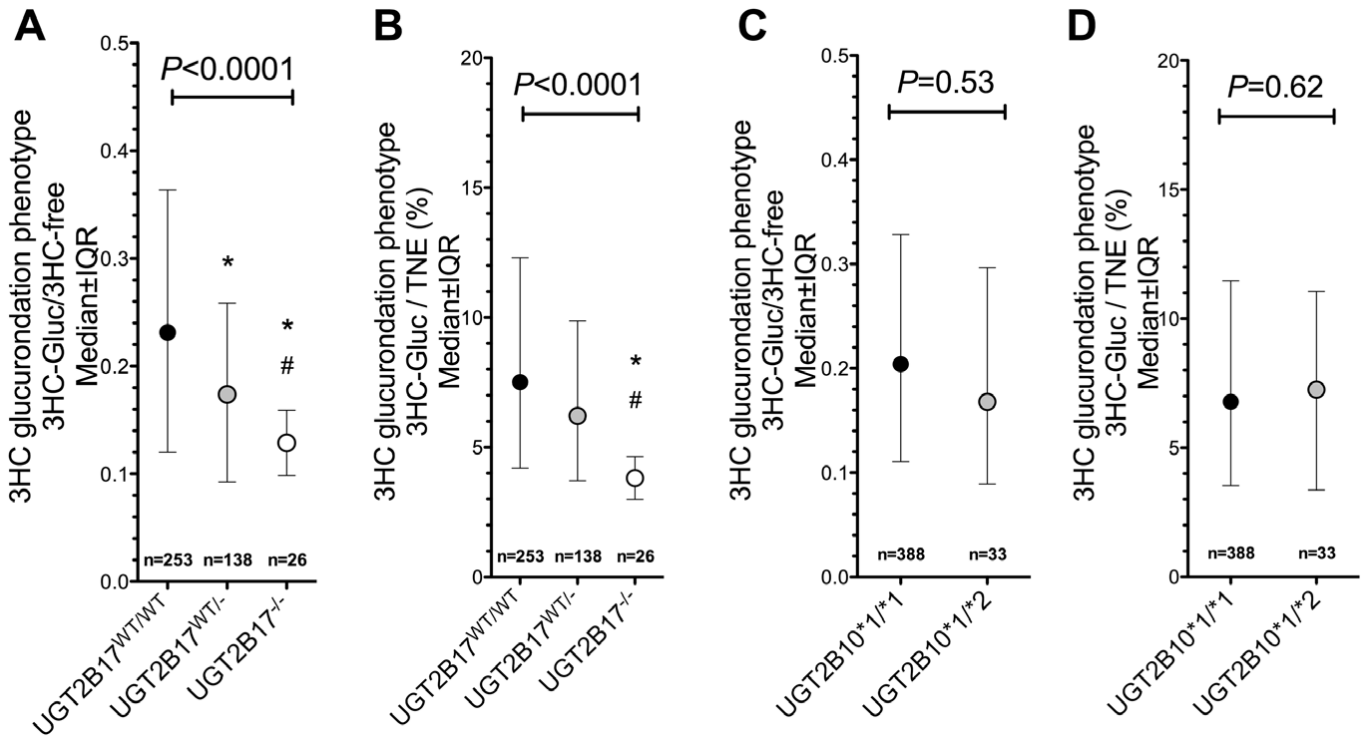
*UGT2B17* gene deletion, but not UGT2B10*2, was associated with a lower 3HC-Gluc phenotype. The *UGT2B17* gene deletion was associated with lower urinary 3HC-Gluc over 3HC-free ratio (**A**) and lower percentage of total nicotine equivalents excreted as 3HC-Gluc (**B**). UGT2B10*2 genotype was not associated with urinary 3HC-Gluc over 3HC-free ratio (**C**) or the percentage of total nicotine equivalents excreted as 3HC-Gluc (**D**). Urinary samples were available for 426 individuals. * = Significantly differ from the UGT2B17^WT/WT^ group. # = Significantly differ from the UGT2B17^WT/−^ group. IQR = Interquartile range. TNE = urinary total nicotine equivalents.

As illustrated by Fig. 1A, the *UGT2B17* gene deletion was associated with lower urinary 3HC-Gluc over 3HC-free ratio, the primary 3HC glucuronidation phenotype, consistent with an impact on 3HC-glucurondation (Fig. 1A, *P*<0.001). Post-hoc analyses revealed that the *UGT2B17* ^WT/WT^ genotype group had a significantly higher urinary 3HC-glucurondation phenotype compared to the *UGT2B17* ^WT/−^ genotype group or the *UGT2B17* ^−/−^ genotype group (medians of 0.23, 0.17, 0.09 respectively, all *P*<0.01) and the *UGT2B17* ^WT/−^ genotype group had a significantly higher urinary 3HC-Gluc over 3HC-free ratio compared to the *UGT2B17* ^−/−^ genotype group (*P*<0.05). The *UGT2B17* gene deletion was also significantly associated with the secondary 3HC glucuronidation phenotype – the percentage of TNE excreted as 3HC-Gluc (Fig. 1B, *P*<0.0001). The median percentages of TNE excreted as 3HC-Gluc were 4.2%, 3.7% and 0.8% for *UGT2B17* ^WT/WT^, *UGT2B17* ^WT/−^, and *UGT2B17* ^−/−^ genotype groups respectively. Post-hoc analyses revealed that the *UGT2B17* ^WT/WT^ genotype group excreted a significantly higher percentage of their TNE as 3HC-Gluc compared to the *UGT2B17* ^−/−^ genotype group (*P*<0.05). The *UGT2B17* ^WT/−^ genotype group excreted a significantly higher percentage of their TNE as 3HC-Gluc compared to the *UGT2B17* ^−/−^ genotype group (*P*<0.05). *UGT2B17* gene deletion was also significantly associated with another 3HC glucuronidation phenotype – the percentage of total 3HC excreted as 3HC-Gluc (Table 1).

### UGT2B10*2 Genotype was not Associated with 3HC-glucurondation Phenotypes *in vivo*

As illustrated by Fig. 1C, UGT2B10*2 genotype was not significantly associated with the primary 3HC-glucurondation phenotype. Furthermore, UGT2B10*2 genotype was not significantly associated with either secondary 3HC-glucurondation phenotypes (3HC-Gluc/TNE and 3HC-Gluc/3HC-Total). The median percentages of TNE excreted as 3HC-Gluc were 6.8% and 7.3% for UGT2B10*1/*1 and UGT2B10*1/*2 individuals respectively (Fig. 1D, non-significant), and approximately 17.4% of total 3HC was excreted as 3HC-Gluc in UGT2B10*1/*1 individuals compared with the 15.9% in UGT2B10*1/*2 individuals (Table 1. non-significant). Thus, we found no evidence of this *UGT2B10* variant altering 3HC glucuronidation *in vivo*.

### *UGT2B17* Gene Deletion was not Associated with Altered Plasma or Urinary NMR

**Figure 2:**
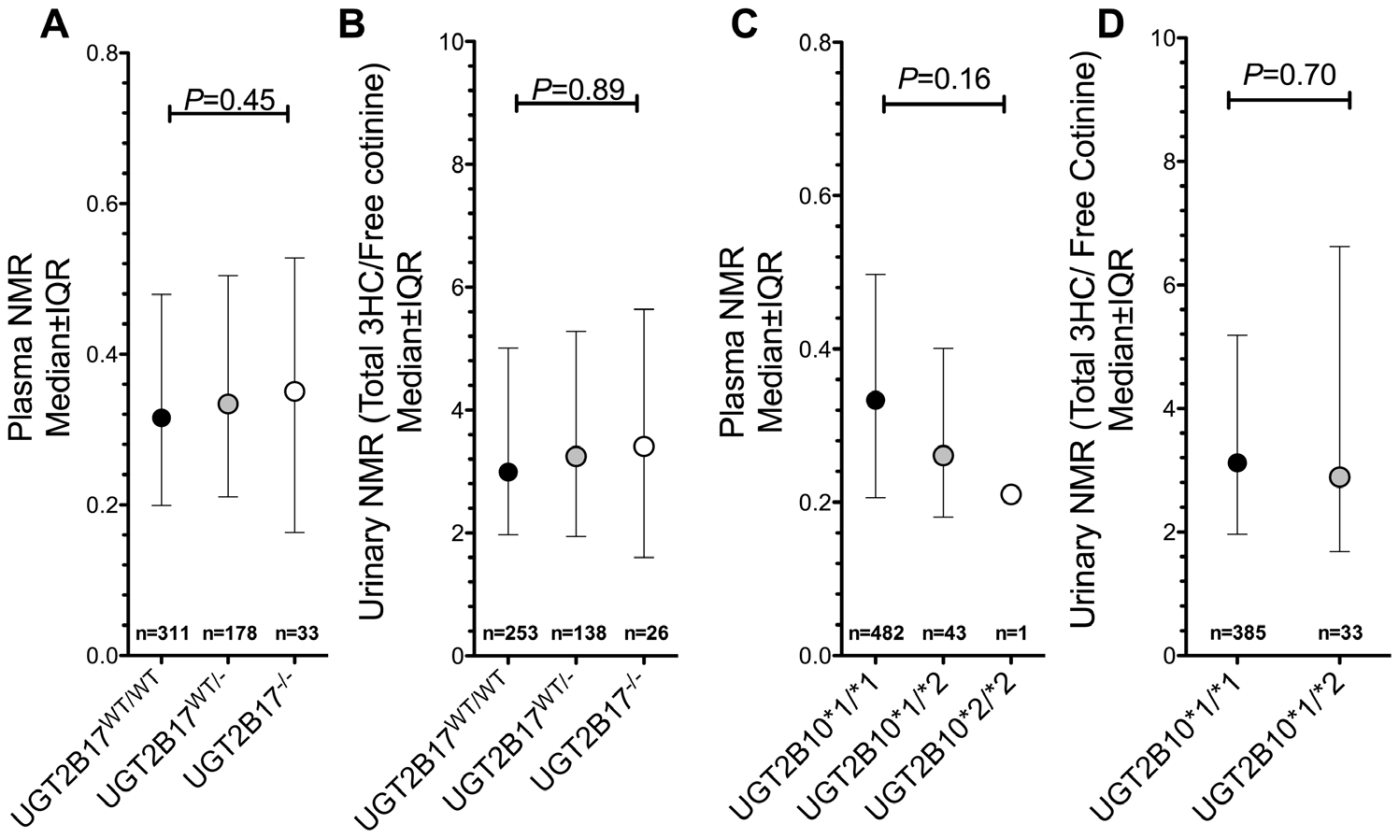
The association between *UGT2B17* gene deletion and UGT2B10*2 with NMR. *UGT2B17* gene deletion was not associated with plasma (**A**) or urinary NMR (**B**). UGT2B10*2 genotype was not associated with plasma (**C**) or urinary NMR (**D**). Urinary samples were only available for 426 individuals. IQR = Interquartile range. No significant difference in plasma NMR between *UGT2B10* genotype groups was found when the UGT2B10*1/*2 and *2/*2 groups were pooled. Similar findings were observed after statistically adjusting for gender, age and body mass index.

As illustrated by Fig. 2A and Fig. 2B, the *UGT2B17* gene deletion was not associated with either plasma or urinary NMR. The median plasma NMR were 0.32, 0.33 and 0.35 for *UGT2B17^WT/WT^*, *UGT2B17^WT/−^* and *UGT2B17^−/−^* respectively (Fig. 2A, non-significant). The median urinary NMR were 3.0, 3.2 and 3.4 for *UGT2B17^WT/WT^*, *UGT2B17^WT/−^* and *UGT2B17^−/−^* respectively (Fig. 2B, non-significant). *UGT2B17* gene deletion was also not associated with the other versions of the urinary NMR, total 3HC/total cotinine or free 3HC/free cotinine (*P* = 0.45 and 0.56 respectively).

### UGT2B10*2 Genotype was not Associated with Altered Plasma or Urinary NMR

As illustrated by Fig. 2C and 2D, UGT2B10*2 genotype was not associated with altered plasma or urinary NMR. UGT2B10*2 was also not associated other versions of the urinary NMR such as total 3HC/total cotinine or free 3HC/free cotinine (*P* = 0.94 and 0.72). No significant differences were observed even when the UGT2B10*1/*2 and UGT2B10*2/*2 genotype groups were combined during these analyses.

### 3HC Glucuronidation Phenotype did not Correlate with Plasma or Urinary NMR

**Figure 3:**
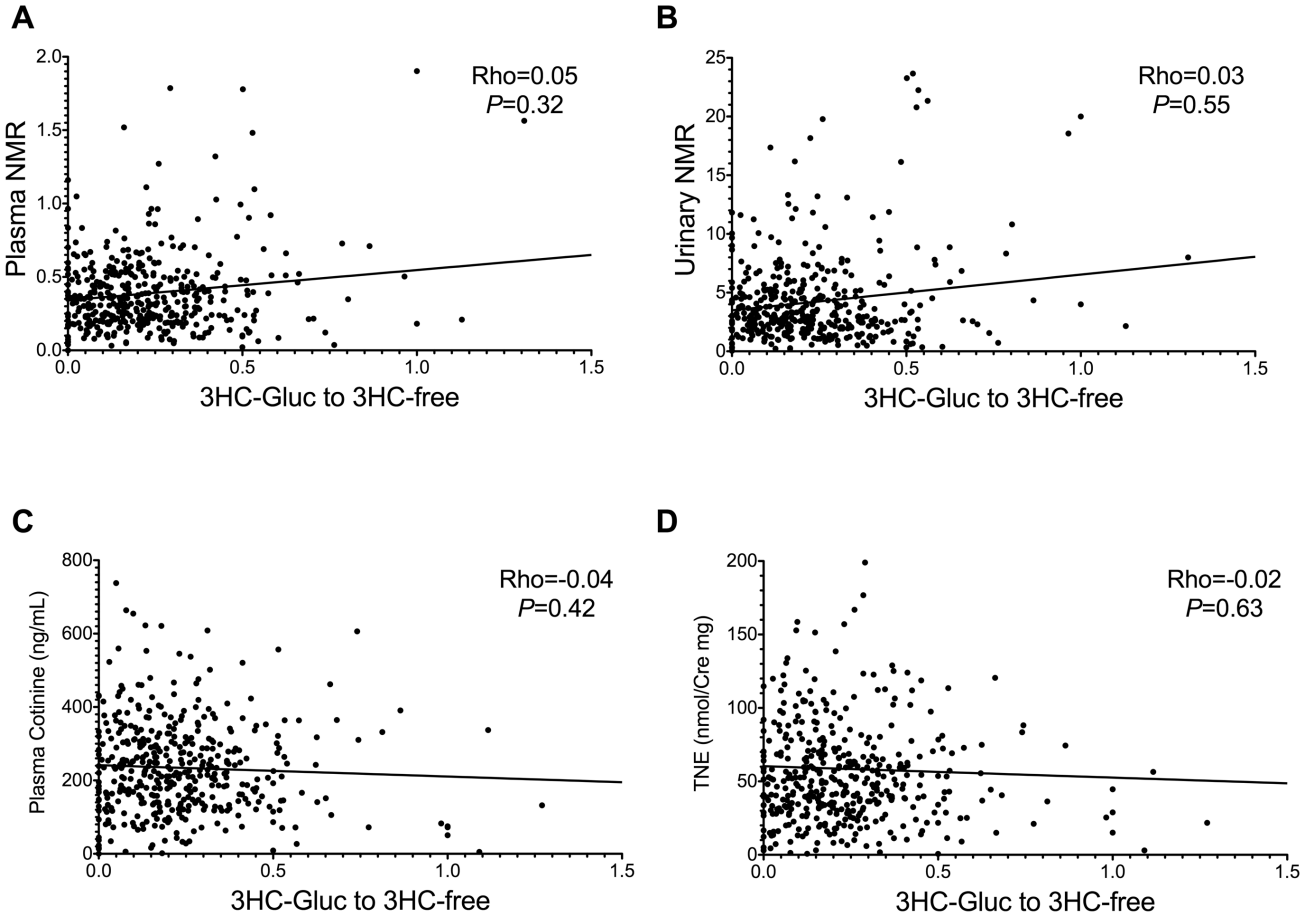
The urinary 3HC-Gluc over 3HC-free ratio did not correlate NMR or nicotine consumption. The urinary 3HC-Gluc over 3HC-free ratio did not correlate with either plasma NMR (**A**) or urinary NMR (**B**). The urinary 3HC-Gluc over 3HC-free ratio also did not correlate with either plasma cotinine (**C**) or the urinary TNE (**D**).

The primary 3HC glucuronidation phenotype (the urinary 3HC-Gluc over 3HC-free ratio) did not correlate with either the plasma (Fig. 3A, Spearman’s Rho = 0.05, P = 0.32) or the urinary (Fig. 3B, Spearman’s Rho = 0.03, P = 0.55) NMR. The primary 3HC glucuronidation phenotype was also not correlated with the alternative versions of urinary NMR, total 3HC/total cotinine or free 3HC/free cotinine (Spearman’s Rho = −0.03 and 0.02; *P* = 0.52 and 0.55 respectively). Furthermore, the urinary 3HC-Gluc over 3HC-Free ratio did not correlate with either plasma cotinine (Fig. 3C, Spearman’s Rho = −0.04, P = 0.42) or the urinary TNE (Fig. 3D, Spearman’s Rho = −0.02, P = 0.63), suggesting variation in 3HC glucuronidation did not change tobacco consumption, consistent with a lack of effect of variation in 3HC glucuronidation on NMR.

## Discussion

Here we established that among African American smokers the *UGT2B17* gene deletion, but not UGT2B10*2 genotype, was associated with slower 3HC glucuronidation *in vivo*. In human liver microsomes, 3HC can be either *O*-glucuronidated on its 3′-hydroxyl group to form 3HC-*O*-Glucuronide or *N*-glucuronidated to form 3HC-*N*-Glucuronide [19]. UGT2B17 exhibited highest 3HC-*O*-glucuronidation activity among all known human UGT1A and UGT2B enzymes [19]. Our observations that *UGT2B17* gene deletion was associated with the 3HC-Gluc over 3HC-free ratio, percentage of TNE excreted as 3HC-Gluc, and the percentage of total 3HC excreted as 3HC-Gluc, are consistent with a predominant role of UGT2B17 in the glucuronidation of 3HC [22]. UGT2B10 exhibits the highest 3HC-*N*-glucuronidation activity among all known human UGT1A and UGT2B enzymes [19]. However 3HC-*N*-Glucuronide is generally not found in urine [25], [35]. Consistent with this we found that UGT2B10*2 genotype was not associated with the 3HC-Gluc over 3HC-free ratio, the percentage of TNE excreted as 3HC-Gluc, or the percentage of total 3HC excreted as 3HC-Gluc suggesting that 3HC-*N*-glucuronidation, via UGT2B10, does not contribute substantially to 3HC-glucuronidation *in vivo*.

Our observations also indicate that neither the *UGT2B17* gene deletion nor the UGT2B10*2 genotype were associated with plasma or urinary NMR. The NMR is used extensively as an *in vivo* indicator of nicotine clearance in studies of smoking behaviors and smoking cessation trials [12]–[14], [36]–[38]. Here we demonstrated that neither *UGT2B17* gene deletion nor UGT2B10*2 genotype significantly altered NMR, suggesting variation in 3HC-glucuronidation is unlikely to alter the relationship between NMR and nicotine clearance or to affect the utility of NMR in smoking studies. In this study, each *UGT2B17* gene deletion allele was responsible for a roughly 5% reduction in the percentage of total 3HC excreted as 3HC-Gluc (see Table 1). Of note, a large percentage of the individuals who were homozygous for *UGT2B17* gene deletion had detectable of 3HC-Gluc levels suggesting other UGTs can also glucuronidate 3HC reducing the likelihood that genetic variation in any one of the UGTs alters this pathway substantially. Together this suggests that genetic variation in *UGT2B17* and/or UGT2B10*2 is unlikely to have a substantial effect on 3HC clearance, 3HC plasma levels or NMR.

Another notable observation was that the urinary 3HC-Gluc over 3HC-free ratio, indicative of 3HC glucuronidation activity (i.e. the total activity of all the enzymes involved in this pathway), was not correlated with plasma or urinary NMR. Here, as seen before [22], [31], only 17% of total 3HC was excreted as 3HC-Gluc suggesting that the majority (>80%) of 3HC is cleared without further metabolism in humans and the glucuronidation of 3HC is a relatively minor pathway in 3HC clearance. Together this indicates that variation in 3HC glucuronidation has a very minor effect on the clearance of 3HC and does not have a meaningful impact on NMR values. In addition, we found that neither genetic variation in *UGT2B17* and *UGT2B10,* nor the rate of 3HC glucuronidation, had an effect on levels of smoking as indicated by cigarettes per day, plasma cotinine levels or urinary TNE, and on levels of nicotine dependence, consistent with the minimum impact on NMR.

Our observations should be interpreted in the context of some potential limitations. This study was conducted in African Americans. It is possible that the *UGT2B17* and *UGT2B10* gene variants could have different magnitudes of effect on 3HC glucuronidation in other racial groups. The prevalence of UGTB10*2 was relatively low in African Americans, but a total lack of effect in 540 people would still argue against it contributing meaningfully in other populations. In addition, our study population reported consuming relatively low numbers of cigarettes per day which may limit the generalizability the findings. However, the observed cotinine levels in this study (mean = 240 ng/mL) suggested the findings should be generalizable to both light and heavy smokers since the 240 ng/mL cotinine level was very similar to those previously observed in Caucasian and African American heavy smokers [30]. The high cotinine levels relative to the level of reported smoking may be explained by both the higher nicotine intake per cigarette and the lower ability to clear cotinine (due to genetic variants in *CYP2A6* gene, resulting in the accumulation of the cotinine) in African American smokers [7], [30].

In conclusion, we demonstrated that the *UGT2B17* gene deletion, but not the UGT2B10*2 genotype, significantly altered 3HC glucuronidation. However, variation in 3HC glucuronidation activity, including these caused by *UGT2B17* gene deletions, did not significantly alter NMR and is unlikely to affect the clinical utility of NMR in smoking behavior and cessation studies.
